# Counterfactuals of effects of vaccination and public health measures on COVID-19 cases in Canada: what could have happened?

**DOI:** 10.3389/fpubh.2023.1173673

**Published:** 2023-05-09

**Authors:** David M. Vickers, John Hardie, Stefan Eberspaecher, Claudia Chaufan, Steven Pelech

**Affiliations:** ^1^Centre for Health Informatics, Cumming School of Medicine, University of Calgary, Calgary, AB, Canada; ^2^Retired, Ottawa, ON, Canada; ^3^Private Practitioner, Ottawa, ON, Canada; ^4^Health Policy and Global Health, Faculty of Health, York University, Toronto, ON, Canada; ^5^Department of Medicine, University of British Columbia, Vancouver, BC, Canada; ^6^Kinexus Bioinformatics Corporation, Vancouver, BC, Canada

**Keywords:** COVID−19, non-pharmaceutical interventions, vaccination, evaluation, simulation modelling

## Introduction

Canada's early response to COVID-19 primarily employed non-pharmaceutical interventions (NPIs)—school and business closures, physical distancing measures, stay at home orders, and mandatory public masking—despite little evidence supporting them (1, 2). These responses were not part of existing pandemic plans (3). Indeed, they ignored the basic principles of pandemic planning, which is to minimise serious illness and deaths, as well as limit societal disruptions (4). Canada and the world need detailed analyses of the effectiveness and the costs of the NPIs used to try to control COVID-19. Such analyses must be separated from politics and evidentially based on comprehensive data sets.

Unfortunately, a recent article entitled “Counterfactuals of effects of vaccination and public health measures on COVID-19 cases in Canada: What could have happened?” published in the Canadian Communicable Disease Report by Ogden et al. (5) contains questionable results that sully the evaluation of these important issues. Instead of performing a much-needed analysis of real-world data, Ogden et al. (5) focus on simulation to postulate how the COVID-19 pandemic would have affected Canada had certain public health measures not been implemented. Phrases like “This study illustrates what may have happened…”, and “Canada could have experienced…” are repeatedly expressed by the authors.

While dynamical models can be useful policy simulators, the insights derived from them can all-too-often become the codified opinions of the modeller (6). In this case, the paper by Ogden et al. (5) is less likely an objective lens into the past than a loss of critical distance from an overly complicated model. In this commentary, we discuss selected authors' assumptions and conclude that they are problematic at-best. We first identify empirically antiquated and conceptually ambiguous justifications supporting the article; second, we critique the model used to substantiate the article's conclusions; and finally, we elaborate on the implications of the authors' position for effective public health policy and human wellbeing in times of crisis.

## The justifications

In the “Introduction”, the authors assert that at the start of the pandemic, humans had no known immunity to SARS-CoV-2 (5). However, if humans were truly immunologically naïve to SARS-CoV-2, then it becomes difficult to explain-away the existence of cross-reactive neutralising IgG antibody (7) or memory T-cells (8, 9) to conserved epitopes between endemic coronavirus and SARS-CoV-2 proteins. These latter T cells will activate, expand, and produce IL-2 and IFN-γ when stimulated (*in vitro*) with cognate antigen (8), which very possibly represents a larger central memory response capable of impacting COVID-19 severity (10). Thus, at the very least, exposure to other human coronaviruses, prior to March 2020, appears able to confer measurable levels of immunity that was not considered—even in sensitivity analyses—by Ogden et al. (5) Similar immune responses to the 1918 strain of influenza existed 90 years later (11), and SARS-CoV-2 is unlikely to be the exception.

The authors also state that, unless the public health measures had been adopted, the consequences for Canadians and for Canada's public health system would have been “dire” (5). This claim, which is predicated on other modelling output (12), assumed that because of no effective therapies, Canada experienced an infection fatality rate (IFR) “approaching” 1%—contrasting it with a much lower IFR of 0.4% for seasonal influenza. While the supporting references used by Ogden et al. (5) estimate the case fatality rate (see their main text references 10, 11, 35), other empirical evidence indicates that the IFR for COVID-19 could be much lower than 1%. For instance, Ioannidis (13) determined that the IFR was 0.15–0.20% globally, and 0.03–0.04% for those under 70 years of age. Canadian-specific estimates reported by Ioannidis were 0.59% (overall) and 0.08% (for people aged < 70 years). People aged >70 years only account for 12.9% of the population in Canada (14). Similarly, results from the COVID-19 Forecasting Team (15) were compatible with unadjusted global IFRs between 0.0054% and 0.43% for people aged < 50 years, while Canadian-specific, age-adjusted cumulative IFRs decreased from 0.54% (in April 2020) and 0.35% (in January 2021). As for the alleged lack of effective therapies, early in 2020 the international literature indicated that early outpatient treatment with multidrug therapy successfully reduced hospitalisation and death, even among populations considered at high risk because of occupation, underlying health conditions, or age (16–20).

The article's “Chronology of the Epidemic and Public Health Measures” justifies the adoption of public health measures based on the “unrestrained SARS-CoV-2 transmission in Italy.” This implies the rampant spread of the infection throughout the entire population. However, Professor Walter Ricciardi, Italy's scientific advisor, corrected this assumption when he noted that “The median age of people infected with SARS-CoV-2 who [were] dying in Italy has been 80 years, and the average age of patients requiring critical care support [was] 67 years” (21). Ricciardi's point on risk-stratification provides an entirely different perspective on the epidemic's characteristics, and if true, nullifies a key justification for the author's model—a virus that if “unrestrained” would be an equal opportunity killer. As well, as shown in their Table 2, Ogden et al. (5) assume that the listed countries have agreed on what constitutes a COVID-19 death. However, they overlook that until recently, a COVID-19 death was anyone dying with, not necessarily from COVID-19 (this was corrected in Ontario March 11, 2022) (22). They also overlook the substantial differences, worldwide, in definitions of COVID-19 deaths (e.g., China likely counts them very differently than Canada).

## Model structure

Ogden et al. (5) constructed an agent-based model (ABM) for understanding any benefits of Canada's pandemic response. ABMs are computational models for simulating the actions and interactions of autonomous agents (such as people) to understand the behaviour of a system and the processes that govern any outcomes of interest (23). Because ABMs maintain distinct information on every individual in a simulated population, their finer-grained nature allows them to represent certain types of activities, relationships, and interventions with greater precision and flexibility than with more traditional differential equation models used to study pathogen transmission (23, 24). However, the granularity of ABMs carries with it an obligation to understand the minutiae that links a model's structure with its behaviour (24). Ogden et al. (5) have circumvented this necessity by filling-in details of people's movements that are exogenous to the structure of the model, rather than being driven by factors or feedbacks that are internal to its current state. This expanded effort in deepening their model has inhibited a critical broadening of the model boundary that will surely limit its validity (6, 24).

For example, the behaviour of the model's agents has an all-or-none relationship with public health measures: when in-place, people changed their behaviour proportionately to the value Oxford Stringency Index; when not in-place, agents maintain their status quo. This forced dependence on government orders is contrary to other research demonstrating that people's inherent fear of infection, alone, will cause them to alter their behaviour rapidly (25), and often before strict rules are in-place (26).

One consequence of the exogeneous drivers of people's behaviour is that two model-derived metrics have diverged substantially from publicly available data. Cumulative case and death rates reported by April 2022 were approximately 9,000 cases and 98 deaths per 100,000 people, respectively (27). When compared to the estimated values in the “observed baseline” scenario in their Table 3, both these empirical values lie outside the 95th percentiles of their modelled counterparts. This directional bias in model output is indicative of structural errors (28) that could distort comparisons between the calibrated (baseline) model and its counterfactual scenarios.

## Over-reliance on counterfactual scenarios

Like all simulation modelling, ABM approaches require that we depart from real-world environments (e.g., workplaces and schools) and create idealised agents and environmental conditions (29, 30). When Ogden et al. discuss Canada's experience with COVID-19 without restrictive measures or vaccination, they failed to note at least two problems. First, “up to 800,000” COVID-19 deaths are greater than any other historic event in Canada over the last 108 years, corresponding to higher per capita death rates than for all Canadian lives lost in World War I, the 1918 influenza pandemic, and World War II (see Table 1) (31–33). Second, the addition of 800,000 deaths would have large consequences for all-cause mortality over the pandemic period, more than doubling it from approximately 640,000 cumulative deaths (34) to 1.4 million. To us, it is an amazing coincidence that all the provincial pandemic responses, which were applied at different times in different sequences (35), could have produced such a large effect that all-cause mortality was reduced to nearly what can be predicted historically—before March 2020—when there was no pandemic and no public health measures, whatsoever [see Figure 5 in Rancourt et al. (36)].

**Table 1:** Per capita Canadian deaths (per 100,000 people) attributed to World War I, II, and the 1918 influenza pandemic compared to the counterfactual modelling scenario “*no public health measures or vaccination*” of Ogden et al. (5).

**Event**	**Deaths (People)**	**Period**	**Population (mid-Period)**	**Death Rate** (per 100,000)^*^	**Refs**.
World War I	60,000	1914–1918	8,001,000	750	39,40
1918 influenza pandemic	50,000	1918–1920	8,311,000	602	39,40
World War II	44,090	1939–1947	11,795,000	374	39,41
Ogden et al. (5) counterfactual	800,000^†^	2020–2022	N/A^#^	2,034 (1,938–2,115)^‡^	5

^*^Death Rate = (Deaths / Population) × 100,000;^†^Upper limit of mortality count for the “no public health measures or vaccination” scenario taken from Table 1 in Ogden et al. (5); ^#^Ogden et al. do not provide an estimate of the Canadian population in their article;^‡^Median (95^th^ percentiles) per capita death rate for 100 model realisations taken from Table 3 in Ogden et al. (5).

## Final comments

It is important that we try to assess the effectiveness and the costs of pandemic interventions dispassionately. The justifications and conclusions in the article by Ogden et al. (5) which are derived from a single model, with no sensitivity analyses around key assumptions, leads us to wonder: what was its point? Was it to showcase an evidence-based analysis of actual policies and their alternatives? Or was it an attempt to justify government action, despite other evidence of their limited benefit (37)?

As academic exercises, counterfactual analyses and what could have happened can possess epistemic benefits. These modes of thought and speech have been the subject of study in philosophy, politics, and history (38), as well as in medical decision making (39). In the analysis by Ogden et al. (5) counterfactuals represent an attractive means for “re-running” history. However, they represent a great disservice to public health in the way they have been used by Canadian public health officials. Just as counterfactual scenarios were used to justify “doing something” during the pandemic (e.g., (40)), the historical revisions of Ogden et al. (5) are used to vindicate that having “done something” was the right course of action. However, unlike modelling the future—which is testable—there is no way to demonstrate whether counterfactual scenarios are right or wrong about “what could have happened.” The historical path, the one involving no interventions, was foreclosed the moment the pandemic responses began (29). Neither Ogden et al. (5) nor anyone else, can ever observe the simultaneous response and non-response of Canada's experience with SARS-CoV-2. Instead, all that remains are “what-if?” statements and modelling output akin to the simulated worlds found in online games; they might appear convincing, but their foundations are imaginary, and their walls are pixel thin (41).

That said, the article by Ogden et al. (5) has two redeeming features. The authors admit that: one, Canada's response to the pandemic was not perfect; and two, that the unintended consequences of the public health measures need to be investigated. It will be a measure of the honesty, courage, and integrity of the Public Health Agency of Canada, and their Provincial partners, if the latter is ever realised.

## Author contributions

DV, SE, JH, CC, and SP contributed to the conception, writing, and editing of this opinion article. All authors contributed to the article and approved the submitted version.
